# Insurance Fraud in Korea, Its Seriousness, and Policy Implications

**DOI:** 10.3389/fpubh.2021.791820

**Published:** 2021-11-15

**Authors:** Jeyong Jung, Byung-Jik Kim

**Affiliations:** ^1^Department of Police Science, University of Ulsan, Ulsan, South Korea; ^2^College of Business Administration, University of Ulsan, Ulsan, South Korea

**Keywords:** insurance fraud, problem diagnosis, national leadership, dynamic concentrated approach, big data technologies

## Abstract

Several characteristics of insurance fraud including its chronic nature justifies the need for identifying feasible proposals which can be expected to bring about significant impacts. Recent statistics show that insurance fraud is now consistently on the increase. However, insurance fraud is highly fragmented and each offence is not significant enough to elicit active interest among the public or interventions from the police. Three problems have been identified and diagnosed. These were a lack of awareness, an absence of a national leadership and also limited attention directed to insurance fraud by the investigating authorities. Based on these, three recommendations have been suggested. (1) Embarking on and developing a national initiative by central government, (2) Taking a dynamic concentration approach to send deterrent threats to potential fraudsters, and (3) Using big data technologies to detect clandestine activities by organised groups.

## Introduction

Insurance fraud is now an entrenched social issue in most countries and requires both proactive responses and prevention measures (1). In Korea, insurance fraud is defined in Article 2 of the ⌜Special Act on the Prevention of Insurance Fraud⌟ as an act of claiming insurance payment by deceiving the insurer with respect to the occurrence, cause, or details of an insurance incident. Insurance fraud has been rampant in the private sector and has now spread to public insurance including health insurance and industrial accident insurance (2). In a situation like this, insurance fraud cases can be said to be serious crimes that potentially threaten all members of society. In addition, insurance fraud crimes such as intentionally causing car accidents or receiving unnecessary treatment for the purpose of claiming insurance payments can do direct damage to the national economy.

Although studies on fraud have been increasing steadily, there is very thin research on insurance fraud globally (3). Since late 1980s, scholars have recognised the significance of the problems and tried to understand the scale, severity, and causes of insurance fraud and to identify ways to deal with such fraud. In particular, Clarke (4) laid the foundation of the domain of insurance fraud by examining the extent of insurance fraud and analysing similarities and differences of national responses. More recently, some researchers started to pay attention to insurance fraud and to identify a specific type of insurance fraud to fill the research gap [e.g., (5, 6)]. However, complex nature of insurance fraud and a lack of empirical data have hampered an accumulation of literature.

There are several clear characteristics to insurance fraud. First, damage is indirect and comprehensive. Insurance fraud may cause initially direct damage to insurance companies, but the damage is ultimately passed on to future policyholders by raising premiums. Second, insurance fraud cases are mostly complex and diverse. It is complex in nature in that some serious types of insurance fraud accompany commission of other types of crimes, such as murder, car accidents, and arson to defraud insurance companies. In addition, insurance covers almost all risks in everyday life, therefore the modus operandi of insurance fraud is diverse. Third, insurance fraud involves collusion. Insurance fraud is often carried out jointly by two or more people to disguise an insurance accident or to free the perpetrator himself/herself from the crime. Fourth, insurance fraud is now becoming much more organised. In recent years, insurance fraud has usually been organised gradually with the emergence of specialised insurance fraud groups involving many people including gangs, hospitals and clinics, maintenance companies, and taxi drivers. These characteristics imply that insurance fraud is quite elusive yet the scale and extent of it looms large in society.

As a summary of insurance fraud in Korea this paper examines first the current situation and circumstances regarding the issue by reviewing recent statistics and trends. Second, it diagnoses associated problems and, third, provides policy options to deal with it. Specifically, the purpose of this paper is to suggest actionable recommendations to the authorities to reduce insurance fraud in Korea. This is now based on a greater awareness of related problems growing in scale and intensity.

## Recent Figures and Statistics

In Korea, the Financial Supervisory Service (FSS) is responsible for responding to insurance fraud. According to the annual report by the FSS, insurance fraud statistics consist of three primary categories. These are the execution of an intentional accident, false claims, and overreporting of damage (by hospitals or car maintenance shops) (7).

The first category refers to executing an accident intentionally to claim an insurance payment, as for example committing suicide, homicide, a car accident, property damage, arson, etc. The most prevalent type is using a vehicle. According to the police, a fraudster in Busan deliberately hit adjacent cars while driving with a total of 37 times from February last year to July this year. He targeted adjacent cars when these were changing lanes or violating the central line or traffic signals. He is accused of taking a total of KRW190 million from insurance companies in the name of settlement money and unresolved repair costs (8).

The second category is exaggerating the scale of an accident and false claiming. A major difference between the first and second category is the initiating time of defrauding. The former begins with plotting and conspiring a commission of a fraud accident while the latter involves initiating fraud activities after an accident occurs. This difference tells us that the former type of insurance fraud is more serious and preparatory, therefore often leading to a large-scale claim.

The last type is overreporting of damage not by individual fraudsters alone, but as a result of collusion between individuals and private entities such as hospitals or car maintenance shops. It is committed in an organised and sophisticated way in that such collusion is involved in perpetrating this type of insurance fraud. Based on the investigation of the FSS, the head of a hospital in Seoul recommended false hospitalisation and surgery to patients who did not need hospitalisation, saying, “I will make it possible to receive a large amount of insurance money.” Then, he defrauded KRW 7 billion by issuing a false diagnosis. Along with this, 110 patients unfairly received KRW 6 billion from insurance companies by recording themselves as inpatients only on the hospital charts, even though they were not hospitalised at all. Also, it was revealed that the family of hospital employees was falsely admitted to the hospital and some senior managers acted as a gatekeeper to arrange and connect fake patients (9).

Table 1 shows that, in 2020, the amount of insurance fraud detected was KRW 898,592 million KRW 898,592 million, and the number of people caught by the authorities was 98,826. Compared to the previous year, these figures increased by 2.0% (KRW 11.7 billion) and 6.8% (6,288 people), respectively. Similarly, the total amount of insurance fraud increased by 12.6% between 2018 and 2020 and the total number of identified insurance fraudsters increased by 24.8% from 79,179 to 98,826 over the same period of time. These changes confirm that insurance fraud is continuously on the rise according to these two criteria (i.e., the total damage, the total number of fraudsters).

**Table 1:** Detected amount by insurance fraud types (Amount unit: KRW 1 million^1^) (10).

	**2018**		**2019**		**2020**	
	**Amount (Percentage)**	**The number of fraudsters (Percentage)**	**Amount (Percentage)**	**The number of fraudsters (Percentage)**	**Amount (Percentage)**	**The number of fraudsters (Percentage)**
Execution of intentional accidents	108,190 (13.6)	6,445 (8.1)	110,148 (12.5)	7,831 (8.5)	138,546 (15.4)	10,225 (10.3)
False claims	581,007 (72.8)	63,128 (79.7)	644,835 (73.2)	75,918 (82.0)	591,445 (65.8)	72.884 (73.7)
Overreporting of damage	53,854 (6.7)	6,318 (8.0)	54,113 (6.1)	5,155 (5.6)	87,816 (9.8)	8,155 (8.3)
Total	798,161 (100)	79,179 (100)	880,912 (100)	92,538 (100)	898,592 (100)	98,826 (100)

Comparing the types of insurance fraud, “false claims” or exaggeration of the scale of accidents accounted for KRW 591.4 billion (65.8%) and “execution of intentional accidents” took KRW 138.5 billion (15.4%) followed by KRW 87.8 billion (9.8%) of “overreporting of damage” or exaggerated claims from hospitals and maintenance companies.

The annual report also indicated that the number of false hospitalizations decreased in 2020 compared to the previous year. This was mainly because hospital beds were in short supply due to Covid-19. Recently, depending on the coverage of the insurance product, organised fraud cases, such as collaborating with brokers, is increasing. More specifically, there is also an increasing number of active forms of insurance fraud that manipulate insurance accidents. This would include receiving unnecessary treatment from hospitals and inflating it to claim insurance money or the purchase excessive insurance for the purpose of defrauding insurance companies.

It might also be noted that the proportion of the detected amount compared to the actual amount of insurance claims was just 1.53%, and which has been declining for some time. The fact that the insurance fraud detection rate has been declining can be interpreted in different ways. It may be that the actual proportion of insurance fraud is decreasing. But, referring to the statistics of the insurance fraud identified above, this scenario is quite unlikely. However, it may be that less insurance fraud cases are now being detected for a number of reasons, and this scenario is more likely. The point is that the ability of both the authorities and insurance companies in terms of detection lag behind the skills of insurance fraudsters. Consequently, a diminishing detection rate leads to higher “dark figures” of offences which are either not detected or not recorded. This can pose a further risk to insurance companies and the government.

If we look at the average fraud amount per fraudster, a clearer picture of the current situation can be identified. Approximately 55.9% of fraud cases involve less than KRW 3 million and the average fraud amount per person is about KRW 9.1 million. Less than KRW 1 million account for 27.8%, less than KRW 3 million takes 55.9%, and less than KRW 5 million takes around 71.2%. About two thirds of insurance fraud cases involved insurance payments claims of less than KRW 5 million. These figures imply that the vast majority of insurance fraud cases can be considered to be low volume crime. Like cyber fraud (11), insurance fraud is largely fragmented and each offence is small enough not to elicit the active interest and intervention of public and investigating authorities.

## Diagnosis of Associated Problems

### Lack of Awareness of Insurance Fraud

There has been lack of research and an absence of government policy as to insurance fraud for two reasons. The first concerns the lack of awareness among the public as to the extent of insurance fraud. Citizens generally do not perceive insurance companies as victims. They think those companies are generally large corporations and they are generating huge profits from citizens with high insurance premiums. Therefore, defrauding insurance companies is not viewed as a great threat to those companies, thus not fitting into a category of serious crime. This is in part because premium increases to future policyholders due to insurance fraud happen over the long term so that people do not recognise this as a serious problem (12). The lack of public awareness is of course also associated with the absence of a national leadership on this issue as is explored below.

### Absence of National Leadership

The second factor involves the mediocre response of the government. The government believes that insurance fraud needs to be dealt with primarily by insurance companies and that these companies have a direct responsibility of managing insurance fraud. As a result of this, national statistics on insurance fraud are still insufficient. Also, government interventions have been taken quite recently only because the government began to realise the significance and urgency of the situation. For example, the^1^ ⌜Special Act on the Prevention of Insurance Fraud⌟ was only passed quite recently in March 2016. In fact, there are several public organisations which are tasked to address insurance fraud. However, these organisations are only loosely connected and do their own work in the absence of any national leadership. It is evidenced by the fact that the higher government (i.e., Prime Minister's Office) has as yet not publicised a national framework to deal with this problem. This does not mean that there is no government agency which responds to insurance fraud. In fact, there are several public entities. Those agencies include the FSS which bears the primary responsibility as a public entity along with criminal justice agencies, such as the police and the Prosecutor's Office (13). The current situation is that insurance fraud is dealt with by them quite separately. Because of this it is fair to say that a national leadership strategy has yet to be formulated and this is also, arguably, a consequence of the lack of public awareness as to the extent of the problem.

### Low Priority Given to Insurance Fraud by Investigating Authorities

The investigation authorities have never made insurance fraud a priority. In general, investigative agencies focus investigative power primarily on either violent crime such as murder, robbery, and sexual assaults associated with organised gangs or on imminent threats such as cyberterrorism, voice-phishing fraud, and cryptocurrency, while demonstrating little interest in insurance fraud (12).

It is evident that the police do not pay much attention to insurance fraud as a separate type of crime. Police statistics provide data on insurance fraud cases which violated ⌜Special Act on the Prevention of Insurance Fraud⌟. In 2020, the number of reported insurance fraud cases to the police were 3,465 and 3,330 perpetrators were arrested with an arrest rate of 96.1% (10). However, this does not encompass the whole number of insurance fraud cases. Fraudsters arrested by the police under the special Act (3,330) in fact account for only 3.4% of the total number of fraudsters detected (98,826) in 2020 (see: Table 1). Indeed the vast majority of insurance fraud cases are charged under Article 347 Fraud of Criminal law. The problem is that insurance fraud charges under the Criminal law are tallied simply as general fraud without any detailed classification. For this reason, the police do not know the real scale of insurance fraud. This statistical issue obscures a landscape of criminal investigation against insurance fraud, which serves to impede the police in providing greater resources to deal with insurance fraud.

## Policy Implications

### Creation of a National Initiative to Coordinate Public and Private Organisations

The increasing trend in insurance fraud indicates that this type of financial crime needs a different approach. Korea is well-known for addressing a type of crime considered as serious and a threat to society by taking when appropriate a national initiative. The national initiative is usually undertaken by the Prime Minister's Office. The Office sets out a national strategy and convenes regular meetings with related private and public organisations to execute the strategy and any subsequent measures. The interdepartmental approach was, for example, adopted for cases such as gang violence, cryptocurrency-related crime, voice-phishing fraud, and property market manipulation (14). In a similar vein, there is the “fraud justice network” in England and Wales which encompasses various criminal and non-criminal justice bodies (15). It is worth noting that non-criminal justice organisations took the substantial role in sanctioning fraudsters. This approach has been recognised as being both effective and efficient in addressing a certain type of crime at the time. However, when it comes to insurance fraud, this approach has not been taken, which implies that insurance fraud is not perceived as serious enough to merit a similar national drive by the higher government. And while these agencies undertake their own work, it is difficult to see or expect these loosely coordinated efforts generating any significant visible outcomes. It is therefore strongly recommended that public and private partnerships are in future driven by the higher government. This would also reflect the fact that the private sector is, undoubtedly, an important stakeholder in insurance fraud.

### Pursuit of Dynamic Concentration to Send Credible Threats to Potential Fraudsters

There are several strategies to control crime such as community policing, evidence-based policing, zero-tolerance approach, hot spot policing, and problem-oriented policing (16). Another strategy worthy of note is an approach known as “dynamic concentration” which was suggested by Mark Kleiman. He argued that targeted and concentrated sanctions against crime is a more effective law enforcement strategy than randomised law enforcement (17). This theory is premised on the idea that the police cannot crack down on all criminal cases and arrest all criminals. It demonstrates how to bring about high deterrence effects with limited manpower and resources. Kleiman (17) suggested that if the authorities send warnings to potential violators and, subsequently, swiftly respond to a few violators with proactive and massive sanctions, potential violators will be convinced that they will face similar sanctions. The importance of sending warnings is also drawn from other studies. Blais and Bacher (18) argued that a written threat reminding the insured of the possible sanctions for insurance fraud had an influence on changing their behaviours. This deterrence effect can occur once the strong message of the authorities reaches a tipping point which can change a high-violation situation to a low-violation one. This approach could be applied to addressing insurance fraud in Korea. Considering the scale, severity, and longevity of insurance fraud, tackling every identifiable case is almost impossible and, therefore, a targeted approach would be more effective. To make it successful two elements need to be checked: (1) Are the authorities ready to concentrate resources? (2) Can the authorities communicate their strong stance to potential violators?

### Use of Big Data Technologies

It is of importance to develop fraud detection capabilities with technological support (19). Many industries and sectors are now aggressively using big data technologies in a way that suits their needs (20). In particular, the banking sector is leading the way. To meet the international and domestic legal compliance banks are trying hard to detect suspicious transactions. The use of big data is necessary for them because humans cannot examine tens of millions of financial transactions occurring every day. Similarly, big data technologies can be a useful tool to spot insurance fraud cases as applications for insurance payments increase over time. It is expected that big data technologies will benefit key stakeholders such as public insurance organisations and private insurance companies by way of being able primarily to detect organised fraud cases. Fraud-related criminal gangs commit fraud offences in a similar fashion with their own members and these activities occur as a criminal network. It is not easy to detect their activities through human “naked” eyes, but may not be difficult to detect them via big data technologies such as network analysis (21). In fact, there have been a few attempts of arresting criminal organisations by using these technologies. However, insurance companies and the government agencies still do not recognise the value and efficiency of these technologies and they are not ready to take advantage of them.

## Conclusion

Insurance fraud is a chronic criminal activity and it has been with us for a long time (22). Although new types and cases of insurance fraud emerge over time, it has not been given the appropriate attention within society and the government. As a social burden and as losses increase, a radical initiative may be required at some point. In this paper, we have investigated insurance fraud and provided three possible actionable recommendations to the Korean government. First, a national initiative needs to be embarked upon by the higher government in a similar way to other major challenges. Second, the “dynamic concentration approach” is specifically recommended in order to send clear deterrent threats to likely fraudsters. Third, it is suggested that full advantage is taken of big data technologies so that the police can identify clandestine activities by criminal groups. Although this article is geographically confined to Korea, discussions and recommendations mentioned here could also be used as reference points to examine situations in other countries.

## Data Availability Statement

The original contributions presented in the study are included in the article/supplementary material, further inquiries can be directed to the corresponding author/s.

## Author Contributions

JJ was the principal investigator and lead the whole process. B-JK was co-researcher and contributed to the article by examining data and writing up some parts. All of them approved the final manuscript for publication.

## Conflict of Interest

The authors declare that the research was conducted in the absence of any commercial or financial relationships that could be construed as a potential conflict of interest.

## Publisher's Note

All claims expressed in this article are solely those of the authors and do not necessarily represent those of their affiliated organizations, or those of the publisher, the editors and the reviewers. Any product that may be evaluated in this article, or claim that may be made by its manufacturer, is not guaranteed or endorsed by the publisher.

## Footnotes

^1^KRW 1 million approximately equals 614 Pound as of early October 2021.

## References

[1] Viaene S Dedene G. Insurance fraud: issues and challenges. Geneva Papers Risk Insurance-Issues Pract. (2004) 29:313–33. 10.1111/j.1468-0440.2004.00290.x

[2] Park Y. A study on the strategies for reducing insurance crime. Korean Police Stud. (2014) 13:61–90.

[3] Gill KM Woolley A Gill M. Insurance fraud: the business as a victim? In: Gill M, editors. Crime At Work. London: Palgrave Macmillan (2005). p. 73–82. 10.1007/978-1-349-23551-3_6

[4] Clarke M. The control of insurance fraud: a comparative view. Br J Criminol. (1990) 30:1–23. 10.1093/oxfordjournals.bjc.a047963

[5] Button M Brooks G Lewis C Aleem A. Just about everybody doing the business? Explaining ‘cash-for-crash' insurance fraud in the United Kingdom. Austral New Zealand J Criminol. (2017) 50:176–94. 10.1177/0004865816638910

[6] Button M Pakes F Blackbourn D. ‘All walks of life': a profile of household insurance fraudsters in the United Kingdom. Security J. (2016) 29:501–19. 10.1057/sj.2013.43

[7] Korea. Financial Supervisory Service. Insurance Fraud Detection Status and Future Plans in 2020. Available online at: https://insucop.fss.or.kr/fss.hpdownload?file=210428_%C1%B6%B0%A3_%28%BA%B8%B5%B5%C0%DA%B7%E1%29_2020%B3%E2+%BA%B8%C7%E8%BB%E7%B1%E2+%C0%FB%B9%DF+%C7%F6%C8%B2+%B9%D7+%C7%E2%C8%C4+%B0%E8%C8%B9.pdf&path=/nws/nbd/ (accessed October 27, 2021).

[8] Ha K. Deliberate Accidents by Targeting a Law-Breaking Vehicle. Two Arrested for Stealing Insurance Money. Newsis. (2021). Available online at: https://newsis.com/view/?id=NISX20211012_0001610170&cID=10811&pID=10800 (accessed October 25, 2021).

[9] Kim H. Even Large Brokers Appear, Insurance Fraud Grows. Ajunews (2021). Available online at: https://www.ajunews.com/view/20211007163954348 (accessed October 28, 2021).

[10] Korea. Financial Supervisory Service. Insurance fraud detection statistics in 2020. Available online at: https://insucop.fss.or.kr/fss.hpdownload?file=%28%BA%B0%C3%B7%29+2020%B3%E2+%BA%B8%C7%E8%BB%E7%B1%E2+%C0%FB%B9%DF%C5%EB%B0%E8.pdf&path=/nws/nbd/ (accessed October 29, 2021).

[11] Loveday B Jung J. A current and future challenge to contemporary policing: the changing profile of crime and the police response. examples of policing fraud in two police jurisdictions: England and Wales and South Korea. Policing. (2021) 15:1633–50. 10.1093/police/paab034

[12] Byeon H Kim S. Current status of insurance fraud and preventive measures in Korea. KIRI Rep. (2019) 462:8–14.

[13] Korea. Financial Supervisory Service. Insurance Fraud Prevention System. Available online at: https://insucop.fss.or.kr/fss/insucop/task02.jsp (accessed October 27, 2021).

[14] Jung J Chun Y. A governmental approach to address risks from cryptocurrencies: focusing on South Korea. Int J Eng Technol. (2018) 7:284–7. 10.14419/ijet.v7i3.24.22664

[15] Button M Shepherd D Blackbourn D. ‘The iceberg beneath the sea', fraudsters and their punishment through non-criminal justice in the ‘fraud justice network' in England and Wales. Int J Law Crime Justice. (2018) 53:56–66. 10.1016/j.ijlcj.2018.03.001

[16] Weisburd D Braga AA. Police Innovation: Contrasting Perspectives. New York, NY: Cambridge University Press (2019) 10.1017/9781108278423

[17] Kleiman MA. When Brute Force Fails. Princeton, NJ: University Press (2009) 10.1515/9781400831265

[18] Blais E Bacher J. Situational deterrence and claim padding: results from a randomized field experiment. J Exp Criminol. (2007) 3:337–52. 10.1007/s11292-007-9043-z

[19] Morley NJ Ball LJ Ormerod TC. How the detection of insurance fraud succeeds and fails. Psychol Crime Law. (2006) 12:163–80. 10.1080/10683160512331316325

[20] Mayer-Schönberger V Cukier K. Big Data–A Revolution That Will Transform How We Live, Think and Work. Boston, MA: Mariner Books (2013).

[21] Jung J. Big data application and management of UK police forces and its policy implications for the Korean Police. J Police Sci. (2019) 19:85–113. 10.22816/polsci.2019.19.1.004

[22] Dornstein K. Accidentally, on Purpose: The Making of a Personal Injury Underworld in America. New York, NY: St. Martin's Press (1996).

